# Increasing phosphorus recovery from dewatering centrate in microbial electrolysis cells

**DOI:** 10.1186/s13068-017-0754-8

**Published:** 2017-03-20

**Authors:** Pengyi Yuan, Younggy Kim

**Affiliations:** Department of Civil Engineering, McMaster University, 1280 Main St. W., JHE 301, Hamilton, ON L8S 4L8 Canada

**Keywords:** Phosphorus recovery, Municipal wastewater treatment, Struvite, Dewatering downstream, Microbial electrochemistry, Cathode structure

## Abstract

**Background:**

Microbial electrolysis cells (MECs) use bioelectrochemical reactions to remove organic contaminants at the bioanode and produce hydrogen gas at the cathode. High local pH conditions near the cathode can also be utilized to produce struvite from nutrient-rich wastewater. This beneficial aspect was investigated using lab-scale MECs fed with dewatering centrate collected at a local wastewater treatment plant. The main objective was to improve phosphorus recovery by examining various cathode configurations and electric current conditions.

**Results:**

The stainless steel mesh (SSM) cathode was relatively inefficient to achieve complete phosphorus recovery because struvite crystals were smaller (a few to tens of micrometers) than the open space between mesh wires (80 µm). As a result, the use of multiple pieces of SSM also showed a limited improvement in the phosphorus recovery up to only 68% with 5 SSM pieces. Readily available organic substrates were not sufficient in the dewatering centrate, resulting in relatively low electric current density (mostly below 0.2 A/m^2^). The slow electrode reaction did not provide sufficiently high pH conditions near the cathode for complete recovery of phosphorus as struvite. Based on these findings, additional experiments were conducted using stainless steel foil (SSF) as the cathode and acetate (12 mM) as an additional organic substrate for exoelectrogens at the bioanode. With the high electric current (>2 A/m^2^), a thick layer of struvite crystals was formed on the SSF cathode. The phosphorus recovery increased to 96% with the increasing MEC operation time from 1 to 7 days. With the high phosphorus recovery, estimated energy requirement was relatively low at 13.8 kWh (with acetate) and 0.30 kWh (without acetate) to produce 1 kg struvite from dewatering centrate.

**Conclusions:**

For efficient phosphorus recovery from real wastewater, a foil-type cathode is recommended to avoid potential losses of small struvite crystals. Also, presence of readily available organic substrates is important to maintain high electric current and establish high local pH conditions near the cathode. Struvite precipitation was relatively slow, requiring 7 days for nearly complete removal (92%) and recovery (96%). Future studies need to focus on shortening the time requirement.

**Electronic supplementary material:**

The online version of this article (doi:10.1186/s13068-017-0754-8) contains supplementary material, which is available to authorized users.

## Background

In conventional wastewater treatment, phosphorus removal is known to be expensive with a large amount of ferric chemical consumption. Biological phosphorus removal also needs large bioreactors to establish anaerobic/aerobic conditions and large pumping capacities to enrich phosphorus accumulating organisms (PAOs) [1, 2]. Another challenge for phosphorus removal in municipal wastewater treatment is the management of downstream wastewater from dewatering processes (i.e., dewatering centrate/filtrate). Such dewatering centrate/filtrate, containing concentrated phosphorus, is often sent back to the mainstream wastewater treatment processes. As a result, phosphorus is continuously recirculated between the mainstream wastewater treatment and sludge treatment systems, making phosphorus removal inefficient in municipal wastewater treatment.

Phosphorus is a valuable resource as it is an essential element in land fertilizers for the agricultural industry and thus closely related to food productivity. Globally mineable phosphorus is owned by a few countries and thus phosphorus production is expected to decrease by the end of the twenty-first century [3], leading to an inevitable drop in food production. Consequently, phosphorus recovery from wastewater has been emphasized in wastewater treatment research so that recovered phosphorus can be used as land fertilizers [4, 5]. While there are a number of methods for phosphorus recovery from nutrient-rich wastewater, such as pyrolysis [6], ion exchange [7], distillation [8], and algae growth [9, 10], here we focused on the struvite precipitation method (MgNH_4_PO_4_∙6H_2_O) for efficient phosphorus recovery from dewatering centrate/filtrate in municipal wastewater treatment. Struvite is a nutrient mineral that can be used as a valuable land fertilizer in the agricultural and landscaping industries.

Struvite precipitation requires substantially high pH conditions [11]; thus, nutrient recovery as struvite often involves consumption of strong base chemicals in conventional precipitation processes (e.g., NaOH) [12–14]. Instead of using base chemicals, microbial electrolysis cells (MECs) can be employed to establish high local pH enough to drive struvite precipitation on the cathode as previously demonstrated [15, 16]. In MECs, organic substrates are oxidized by exoelectrogenic bacteria at the bioanode and water is reduced to hydrogen gas at the cathode by applying an electric voltage between 0.13 and 1.23 V [17–21]. The cathode reaction (2H_2_O + 2e^−^ → H_2_ + 2OH^−^) releases hydroxyl ions, establishing high local pH near the cathode. The high local pH condition has been utilized in a number of MEC studies to enhance precipitation of various chemicals, including toxic heavy metals [22] and struvite crystals [15, 16, 23–25] without adding any base chemicals. Thus, compared to the conventional chemical precipitation methods, MECs can produce struvite without using base chemicals. In addition to struvite production, MECs allow energy recovery in the form of hydrogen gas and organic removal in the wastewater.

Since struvite is crystalized on cathode surfaces, the cathode configuration plays an important role in efficient struvite production in MECs [15]. While a mesh type cathode was found to be more effective than a plate-type cathode in a previous proof-of-concept study with a relatively small amount of struvite crystals attached on the cathode [15], a plate-type cathode can be more efficient than the mesh type if an excessive amount of struvite crystals is created and deposited on MEC cathode surfaces. Also, struvite production in MECs was demonstrated mainly using synthetic solutions [15, 16, 23]. Thus, potential limitations involved in using real wastewater (e.g., low concentration of readily available organic substrates) were not investigated in the previous studies [15, 16, 23–25]. As a result, even though MEC cathodes are proven to drive struvite crystallization from synthetic solutions, there is still a research gap for efficient phosphorus recovery from real wastewater. In this study, we examined dewatering centrate from a local wastewater treatment plant and we also focused on demonstrating complete phosphorus recovery in MECs. To achieve efficient phosphorus recovery, various cathode configurations (mesh vs. foil types; single vs. multiple pieces) were examined in lab-scale MEC experiments for efficient struvite precipitation. In addition, for effective struvite production, high electric current is desired and thus a readily available organic substrate is necessary for MEC operation. Since dewatering centrate is lacking readily available organic substrates, we studied the effect of electric current generation in MECs on the phosphorus recovery. Finally, the time requirement for struvite precipitation was also investigated in this study.

## Methods

### Reactor design and construction

**Fig. 1:**
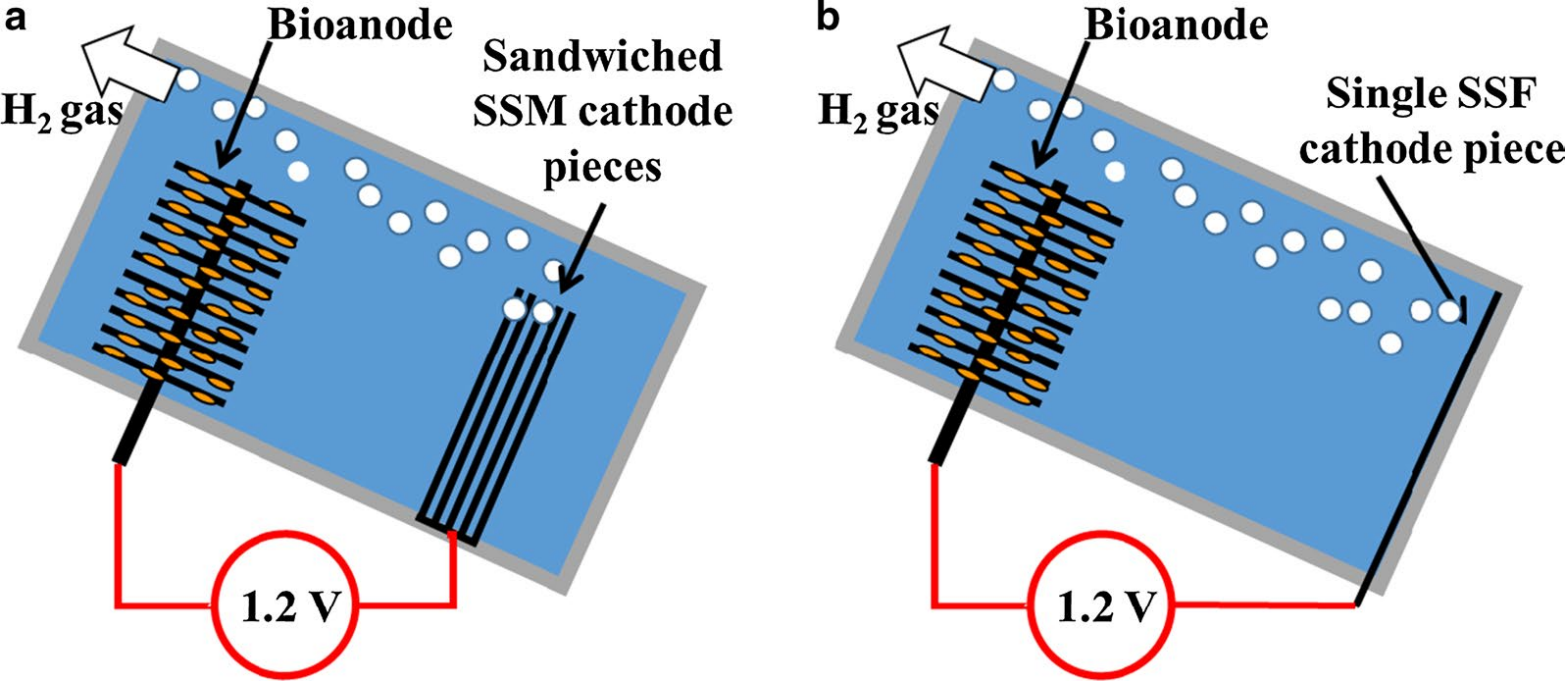
**a** Schematic diagram of MEC constructed with the SSM cathode (5 SSM pieces). **b** Schematic diagram of MEC with the SSF cathode

Single-chamber MEC reactors were built with polypropylene blocks and rubber gaskets with a cylindrical inner space (50 mL; 7-cm^2^ cross section). The bioanode was a graphite fiber brush (2.5 cm in diameter, 2.0 cm long; Mill-Rose, OH), which was heat treated in a muffle furnace at 450 °C for 30 min [26]. To examine the effect of cathode configuration on phosphorus recovery, stainless steel mesh (SSM) and stainless steel foil (SSF) were used as the cathode in the MEC. Since struvite is precipitated on cathode surfaces, the amount of cathode surface areas was examined with the SSM cathode (1, 3, or 5 pieces) (Fig. 1a) (6.3-cm^2^ cross section; McMaster Carr; 304 stainless steel woven wire cloth; 200 × 200 mesh; 0.053 mm wire diameter) while a single piece of the SSF cathode was located in the MEC reactor (Fig. 1b) (7.0-cm^2^ cross section; Trinity Brand Industries, Inc.; 0.0254 mm thickness). The mesh size (200 × 200) was selected as it produced the highest electric current compared to other commonly available stainless steel mesh sizes (e.g., 50 × 50 or 100 × 100). For the 3- and 5-piece SSM cathode, the distance between the SSM pieces was maintained at ~1 mm using a rubber gasket. The MECs were inclined so that created struvite precipitants can be deposited on the cathode and produced hydrogen gas can be easily removed from the reactor (Fig. 1). While energy recovery as hydrogen is an important aspect of MEC studies, we focused more on nutrient recovery and wastewater treatability of MECs in this study.

### Reactor start-up and operation

The MECs were inoculated using effluent from an existing MEC. After the start-up operation using acetate as the substrate, the MECs were operated in fed-batch mode using dewatering centrate collected at a local municipal wastewater treatment plant (Additional file 1: Figure S1). Ammonia concentration in the dewatering centrate was 65.7 ± 2.8 mM and phosphate concentration was 0.43 ± 0.03 mM. The ammonia concentration was sufficiently higher than phosphate because struvite precipitation (MgNH_4_PO_4_) requires the same molar ratio among magnesium, ammonia, and phosphate. The local wastewater treatment plant was operated as conventional activated sludge without biological phosphorus removal; thus, the phosphate concentration in the dewatering centrate could have been higher if biological phosphorus removal had been employed in the mainstream wastewater treatment. Thus, an extra amount of phosphate (1.5, 3.0, or 4.5 mM as Na_2_HPO_4_) was added to the dewatering centrate to simulate downstream wastewater from biological phosphorus removal processes. Note that 80% of phosphorus removed from biological phosphorus removal processes was released in anaerobic digestion [27] and the released amount of phosphate can be as high as 2.6 mmol/g-MLSS (mixed liquor suspended solids) [28]. Thus, we examined various phosphate concentrations in experiments by adding Na_2_HPO_4_ (Table 1). For proper struvite precipitation, 2 mM of MgCl_2_ was added in the dewatering centrate for the all experimental sets. To investigate the effect of electric current on struvite production, NaCH_3_COO addition was also examined in the MEC operation (Table 1).

**Table 1:** Feed preparation and MEC operation in four experimental sets

Set A	1, 3, 5 SSM cathode pieces No phosphate addition Mg:NH_4_:PO_4_ = 2.0:66:0.43 (mM) No acetate addition
Set B	1, 3, 5 SSM cathode pieces 1.5 mM Na_2_HPO_4_ addition Mg:NH_4_:PO_4_ = 2.0:66:1.93 (mM) No acetate addition
Set C	Single SSM cathode piece 1.5, 3.0, 4.5 mM Na_2_HPO_4_ addition Mg:NH_4_:PO_4_ = 2.0:66:1.93–4.93 (mM) No acetate addition
Set D	Single SSF cathode piece 1.5 mM Na_2_HPO_4_ addition Mg:NH_4_:PO_4_ = 2.0:66:1.93 (mM) 12.2 mM NaCH_3_COO addition

Four sets of experiments (Sets A, B, C, and D) were conducted in this study. Sets A and B were designed to investigate the effect of the number of the SSM cathode pieces. In Set C, we studied the effect of various phosphate concentrations assuming biological phosphorus removal processes in the mainstream wastewater treatment. Even though struvite precipitation needs the same molar concentration for Mg^2+^, NH_4_^+^, and PO_4_^3−^, we hypothesized that the kinetics of struvite precipitation can be enhanced by high phosphate concentration. Set D was conducted to improve the struvite recovery using the SSF cathode and high electric current by adding NaCH_3_COO (Table 1). The addition of the NaCH_3_COO did not alter pH of the dewatering centrate, indicating that the dewatering centrate has a sufficient amount of alkalinity.

The applied voltage was 1.2 V using an external power supplier to maximize the electric current in the MEC (GPS-1850D; GW Instek, Taiwan). The electric current was computed by monitoring the voltage crossing an external 10-Ω resistor every 20 min using a digital multimeter and data acquisition system (Model 2700, Keithley Instruments, OH). All experiments were conducted in an air-conditioned laboratory (22.5 ± 0.2 °C).

### Experimental measurement

For each fed-batch cycle, the feed and effluent samples were examined for total phosphorus, ammonia, and COD (chemical oxygen demand) in accordance with the standard methods (Hach Co., CO) [27]. The experimental samples were also analyzed for pH and conductivity (SevenMulti, Mettler Toledo Group, Switzerland). The conductivity of the dewatering centrate was ~8.4 mS/cm and it increased slightly to ~8.6 mS/cm during the MEC operation. The feed pH was ~7.6 and the effluent pH was ~8.2.

For Sets A and B, the SSM cathode was taken from the MEC reactor after 3 fed-batch cycles and the struvite crystals deposited on the cathode were scraped and dissolved in an acid solution (10 mM HCl) to determine the amount of phosphorus recovered as struvite. The MEC operation over three consecutive fed-batch cycles without replacing the cathode allowed investigating the effect of struvite accumulation at cathode surfaces on electric current generation in the MEC. For Sets C and D, the cathode was taken every fed-batch cycle to quantify the precipitated struvite crystals. The phosphorus removal was determined based on the feed and effluent concentration of phosphorus. The amount of phosphorus recovered as struvite was compared with that removed during the MEC operation to determine phosphorus recovery (*r*) as:

1 $$r = \frac{{M_{\text{P}} }}{{nV(c_{\text{feed}} - c_{\text{eff}} )}}$$

*M*_p_ is the total moles of phosphorus in struvite precipitants scraped from the cathode, *n* is the number fed-batch cycles (3 for Sets A and B; 1 for Sets C and D), *V* is the volume of the MEC reactor (0.050 L), *c*_feed_ is the molar concentration of phosphorus in the feed, and *c*_eff_ is the phosphorus concentration in the effluent. The precipitated crystals on the cathode were also analyzed in scanning electron microscopy (SEM) and energy-dispersive X-ray spectroscopy (EDS) to examine the crystal morphology and identification (JEOL JSM-6610LV, Japan). The EDS analysis results confirmed that the precipitants on the MEC cathode were struvite (Additional file 2: Figure S2).

The Coulombic efficiency (CE) was determined by dividing the amount of electrons measured in electric current by the amount of electrons that can be yielded from substrate oxidation as [20]:

2 $${\text{CE}} = \frac{{8\mathop \int \nolimits I{\text{d}}t}}{{FV\Delta {\text{COD}}}}$$

*I* is the electric current, *F* is the Faraday constant, and ΔCOD is the COD removal over a fed-batch cycle. The electric energy requirement (*W*_E_) was calculated using [20]:

3 $$W_{\text{E}} = \mathop \int \nolimits (IE_{\text{ap}} - I^{2} R_{\text{ext}} ){\text{d}}t$$

*E*_ap_ is the applied voltage (1.2 V) and *R*_ext_ is the external resistor (10 Ω).

## Results and discussion

### SSM cathode for struvite production

The phosphorus removal during each fed-batch cycle was consistently high (69–85%) with the SSM cathode in Sets A and B (Table 2). No clear correlation was found between the phosphorus removal and number of SSM pieces, indicating that the total surface area of the SSM cathode did not limit the phosphorus removal by struvite precipitation. In Sets A and B, struvite precipitants were obtained on the cathode after 3 fed-batch cycles and the majority of the precipitants were found on the SSM piece located close to the bioanode.

**Table 2:** Phosphorus removal and recovery (*n* = 3; mean ± standard error)

Experiment	Number of SSM cathode	Phosphorus in feed (mM)	Phosphorus in effluent (mM)	Removal (%)	Recovery as struvite (%)
Set A	1 piece	0.43 ± 0.03	0.13 ± 0.01	69.7 ± 0.8	54.0
	3 pieces		0.13 ± 0.01	70.6 ± 0.5	55.6
	5 pieces		0.13 ± 0.01	69.3 ± 0.5	68.2
Set B	1 piece	1.28 ± 0.09	0.23 ± 0.01	82.4 ± 0.2	10.3
	3 pieces		0.26 ± 0.04	80.0 ± 2.8	15.7
	5 pieces		0.20 ± 0.03	84.7 ± 1.1	26.8
Set C	1 piece	1.36	0.18	87.0	6.8
		2.63	0.37	86.0	6.1
		3.26	0.70	78.6	22.5

By comparing the amount of phosphorus in the deposited struvite crystals with that removed during the MEC operation (Eq. 1), the phosphorus recovery in Set B was relatively lower at 10–27% than that in Set A (54–68%). This drop in the phosphorus recovery with the increased phosphate concentration in Set B indicates that the SSM cathode has a limited capacity to hold produced struvite crystals. In addition, the phosphorus recovery showed increasing trends for both Sets A and B with the increasing number of cathode pieces (Table 2). The increased surface area of the SSM cathode did not affect the phosphorus removal but improved the phosphorus recovery. However, the phosphorus recovery was below 27% especially when an additional phosphate was provided in the dewatering centrate.

Three different phosphate concentrations (1.36, 2.63, and 3.26 mM) were examined in the experimental Set C to simulate downstream wastewater from enhanced biological phosphorus removal processes [28, 29]. The phosphorus removal efficiency was maintained high at 78–87% with only single SSM piece as the cathode (Table 2). Thus, the single-piece SSM cathode was sufficient to remove phosphate for the examined concentrations. However, the phosphorus recovery as struvite crystals on the cathode was insufficient and varying a wide range between 6 and 20% (Table 2).

The difference between the high removal and low recovery can be explained by relatively small struvite crystals on the cathode. The SEM images showed that the majority of struvite crystals are smaller than 10 µm (Additional file 3: Figure S3). As a result, struvite crystals were easily lost through the open mesh spaces when the MEC reactors were disassembled to collect precipitated struvite crystals. Note that the SSM cathode had much larger open spaces between woven wires (80 µm × 80 μm) than produced struvite crystals (a few to tens of micrometers). Thus, the SSM cathode was effective to drive struvite precipitation as previously proven [15]; however, it was not ideal for holding precipitated crystals especially when the cathode was practically covered by struvite precipitants (Additional file 4: Figure S4).

### Electric current and COD removal

**Fig. 2:**
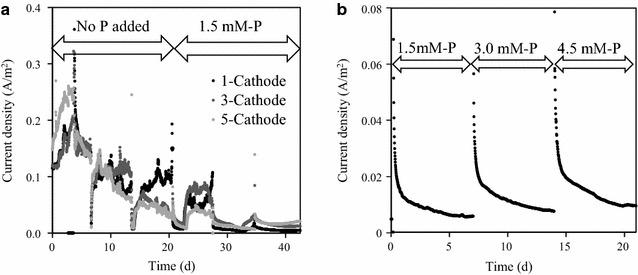
Electric current generation in MECs with the SSM cathode: **a** effect of the number SSM pieces in Sets A and B; **b** effect of phosphate concentration in Set C

There was no clear correlation between the electric current and number of SSM cathode pieces (Fig. 2a), indicating that the electric current generation was not limited by the cathode. The electric current density was mostly below 0.2 A/m^2^ (Fig. 2). As a result, the COD removal was relatively low, varying over a wide range from 15 to 39% (Table 3). The limited COD removal as well as the low electric current can be explained by limited amounts of readily available organic substrates in the dewatering centrate for exoelectrogens. Note that concentration of acetic acid or other volatile fatty acids is relatively low in sludge treated in healthy anaerobic digesters [30–32]. Since the MEC operation was limited by low readily available organic substrate concentration, the increased cathode surface area did not effectively increased the electric current with 1, 3, and 5 cathode pieces (Fig. 2a). Thus, the relatively high COD in the dewatering centrate (487–800 mg COD/L) was not favorably utilized by exoelectrogenic microorganisms at the bioanode. As a result, the CE was relatively low and varying widely from 3 to 86% (Table 3). In addition to the large open area of SSM, the low electric current was also considered to have resulted in the limited struvite recovery (Table 2) because sufficiently high local pH near the cathode was not established due to slow creation of hydroxyl ions (i.e., slow consumption of proton ions).

**Table 3:** COD removal and Coulombic efficiency (*n* = 3; mean ± standard error)

Experiment	Number of SSM cathode	COD in feed (mg/L)	COD in effluent (mg/L)	COD Removal (%)	Coulombic efficiency (%)
Set A	1 piece	600 ± 21	437 ± 64	26.9 ± 11.1	86.3 ± 64.6
	3 pieces		367 ± 12	38.6 ± 4.2	29.9 ± 6.5
	5 pieces		457 ± 30	24.0 ± 3.1	54.2 ± 17.4
Set B	1 piece	487 ± 35	407 ± 22	16.2 ± 2.3	10.4 ± 1.0
	3 pieces		397 ± 27	18.4 ± 1.3	26.7 ± 15.1
	5 pieces		367 ± 43	24.5 ± 7.7	18.7 ± 15.6
Set C	1 piece	730	460	37.0	2.5
		630	480	23.8	5.5
		800	680	15.0	9.7

### Enhanced struvite recovery with SSF and high electric current

**Fig. 3:**
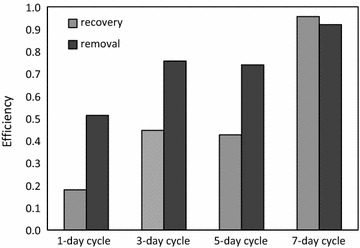
Phosphorus removal and recovery with the SSF cathode (Set D)

**Fig. 4:**
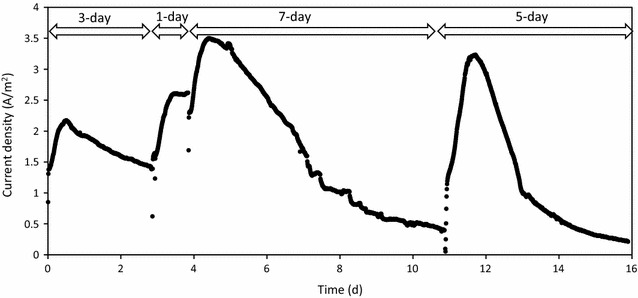
Electric current generation in during the MEC operation with the SSF cathode (Set D)

To improve the struvite recovery, the SSF cathode was used in the MEC and high electric current was induced by adding acetate in the dewatering centrate in Set D. The phosphorus removal was 53% in only 1 day and it increased to 92% in 7 days (Fig. 3). The phosphorus recovery substantially improved from 18 to 96% with the longer MEC operation (Fig. 3). This result indicates that high electric current and a foil-type cathode are necessary to maintain high phosphorus recovery. The high electric current density helped to maintain high local pH near the cathode with a high rate of the hydroxyl ion release from water electrolysis (Fig. 4). Also, the phosphorus recovery as the cathode precipitant was usually lower than the removal (Fig. 3). The phosphorus uptake by microorganisms can potentially explain the discrepancy between the removal and recovery; however, a more systematic approach is necessary to quantify the contribution by microbial uptake in future studies.

In the previous studies, a mesh type cathode worked better for phosphorus removal (40% removal) compared to a foil-type cathode (26% removal) [15]. However, when the amount of phosphorus recovered is substantially large, the SSF cathode resulted in high removal and recovery in this study. Note that the SSF cathode was fully covered with struvite salts in this study (Additional file 5: Figure S5). In a separate experiment (not shown), we also operated the MEC with SSM cathode and acetate; however, the phosphorus recovery was not as high as that with SSF and acetate, indicating that the cathode structure is more important than the presence of a readily available organic substrate.

### Energy requirement for struvite production

The electric energy consumption was 843 J (2.34 × 10^−4^ kWh) for the operation of the MEC with the SSF cathode over 7 days (Eq. 3). Based on this energy consumption, 4.95 MJ (13.8 kWh) is estimated to be necessary to produce 1 kg struvite from dewatering centrate. Similarly, the energy requirement was 1.09 MJ (0.30 kWh) per 1 kg struvite production without adding acetate in Set C. Note that the energy recovered as H_2_ gas was not considered in the energy estimation; thus, the net energy requirement will be substantially smaller as previously discussed [15]. Considering the relatively low energy requirement and enhanced phosphorus recovery, MECs have strong potential for struvite production from nutrient-rich wastewater streams.

## Conclusions

The SSM and SSF cathodes were examined in lab-scale MECs to improve the phosphorus recovery from dewatering centrate. The SSM cathode was effective to remove phosphorus via struvite precipitation, but the phosphorus recovery was insufficient (maximum 68%) because the open space between woven wires (80 µm × 80 µm) was much larger than the size of struvite crystals (a few to tens of micrometers). As a result, the phosphorus recovery was not sufficiently improved by increasing the surface area of the cathode with 5 SSM pieces.

The dewatering centrate from anaerobic digesters contained a small amount of readily available organic substrates for exoelectrogenic bacteria at the bioanode. As a result, the electric current was substantially low in the MEC reactors, resulting in slow water electrolysis at the cathode. Consequently, the local pH near the cathode was not sufficiently high, leading to the limited recovery of struvite from the dewatering centrate. Thus, readily available organic substrates need to be provided in MECs for efficient recovery of phosphorus as struvite.

The SSF cathode was then examined to minimize potential losses of small struvite crystals and acetate was added in the MEC operation as a readily available organic substrate. The high electric current density (>2 A/m^2^ for peak currents) and foil-type cathode resulted in successful struvite production from dewatering centrate with 92% removal and 96% recovery. The phosphorus removal and recovery efficiencies increased with the increasing fed-batch cycle period. A retention time of 7 days was necessary to achieve complete removal and recovery of phosphorus in the SSF MEC. MECs have a potential for struvite production in municipal wastewater treatment plants with a relatively small electric energy requirement: 13.8 kWh per kg struvite production with acetate and 0.30 kWh without acetate. While we demonstrated high energy efficiency and enhanced phosphorus recovery from a real wastewater stream, the purity of the struvite precipitants was not examined in this study. Various MEC operation conditions need to be investigated for their effects on the purity of struvite crystals in future studies.

## Additional files

**Additional file 1: Figure S1** Dewatering centrate collected at a local municipal wastewater treatment plant.

**Additional file 2: Figure S2** (A) EDS analysis results for precipitants recovered from the MEC operation (Set C). (B) EDS analysis results for pure struvite (99.999% purity).

**Additional file 3: Figure S3** SEM images of struvite crystals on the SSM cathode after one fed-batch cycle (Set C).

**Additional file 4: Figure S4** SSM cathode after one fed-batch operation (4.5 mM phosphate addition in Set C).

**Additional file 5: Figure S5** Struvite precipitants on the SSF cathode after 5-day MEC operation (Set D).

## Abbreviations

CE: Coulombic efficiency
COD: chemical oxygen demand
*E*_ap_: applied voltage
EDS: energy-dispersive X-ray spectrometry
MEC: microbial electrolysis cell
PAO: phosphorus accumulating organism
SEM: scanning electron microscopy
SSF: stainless steel foil
SSM: stainless steel mesh
*W*_E_: electric energy requirement for MEC operation
