# Supplementary figures and images for: Increasing phosphorus recovery from dewatering centrate in microbial electrolysis cells
Source: Biotechnol Biofuels. 2017 Mar 20;10:70. doi: 10.1186/s13068-017-0754-8 (PMC5359864; doi:10.1186/s13068-017-0754-8)

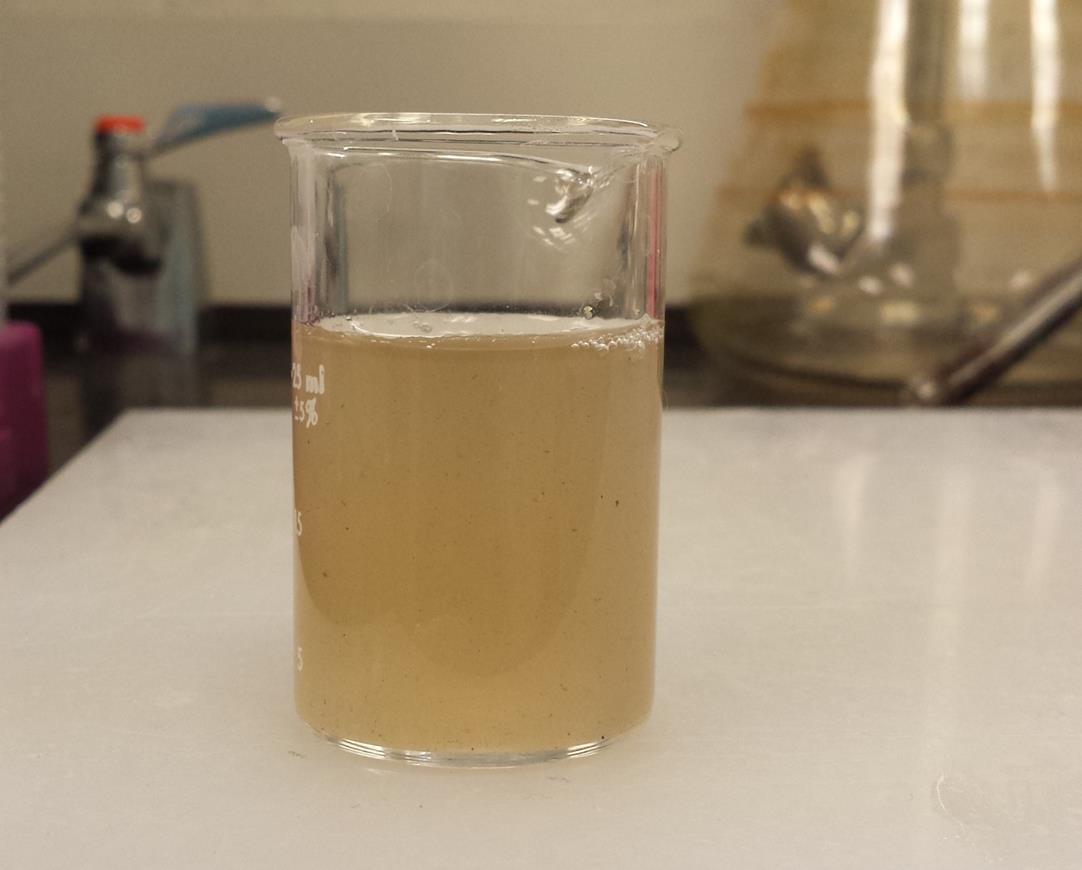


**Figure S1 Dewatering centrate collected at a local municipal wastewater treatment plant.**

Supplement: Supplementary file 1 — Additional file 1: Figure S1 Dewatering centrate collected at a local municipal wastewater treatment plant. [file 13068_2017_754_MOESM1_ESM.docx]

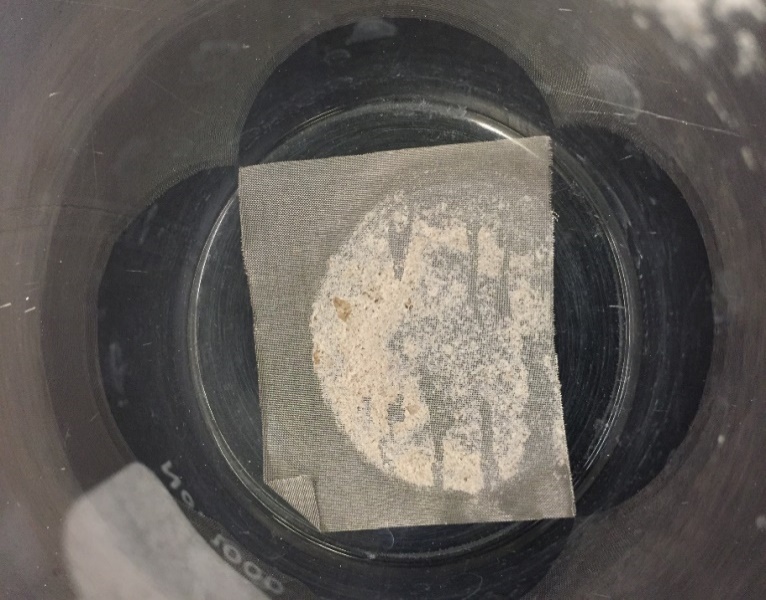


**Figure S4 SSM cathode after one fed-batch operation (4.5 mM phosphate addition in Set C).**

Supplement: Supplementary file 4 — Additional file 4: Figure S4 SSM cathode after one fed-batch operation (4.5 mM phosphate addition in Set C). [file 13068_2017_754_MOESM4_ESM.docx]

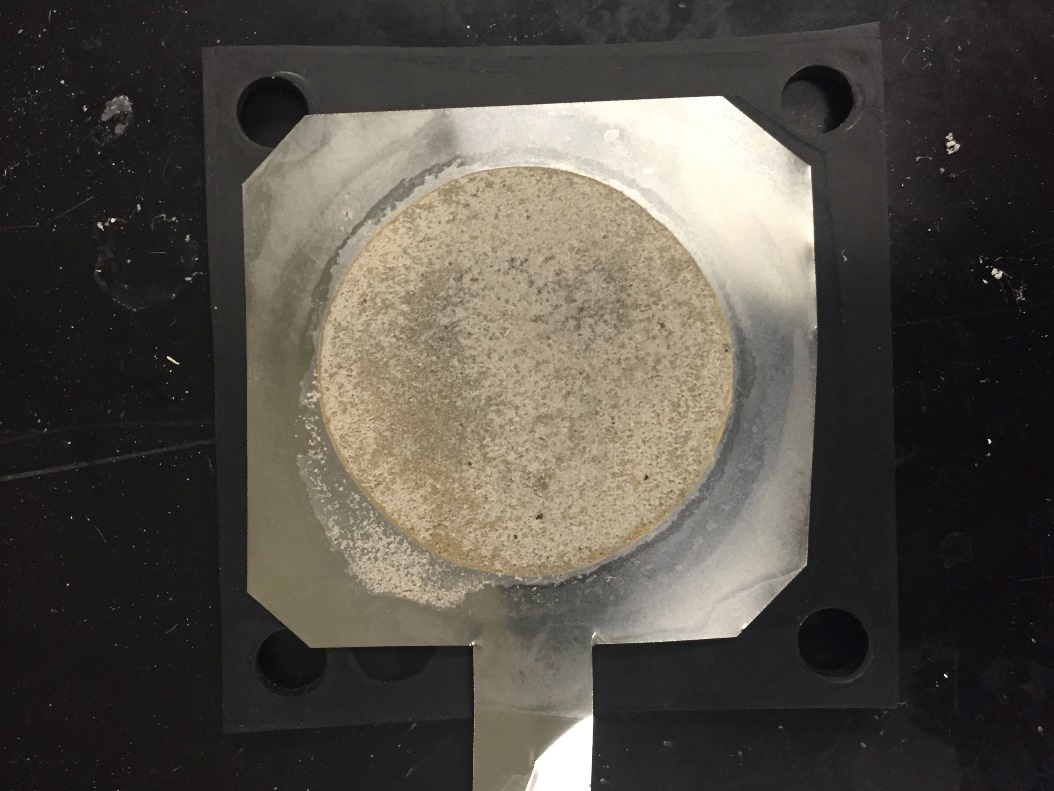


**Figure S5 Struvite precipitants on the SSF cathode after 5-day MEC operation (Set D).**

Supplement: Supplementary file 5 — Additional file 5: Figure S5 Struvite precipitants on the SSF cathode after 5-day MEC operation (Set D). [file 13068_2017_754_MOESM5_ESM.docx]
