# Supplementary material for: Increasing phosphorus recovery from dewatering centrate in microbial electrolysis cells
Source: Biotechnol Biofuels. 2017 Mar 20;10:70. doi: 10.1186/s13068-017-0754-8 (PMC5359864; doi:10.1186/s13068-017-0754-8)
Supplement: Supplementary file 2 — Additional file 2: Figure S2 (A) EDS analysis results for precipitants recovered from the MEC operation (Set C). (B) EDS analysis results for pure struvite (99.999% purity). [file 13068_2017_754_MOESM2_ESM.docx]

**
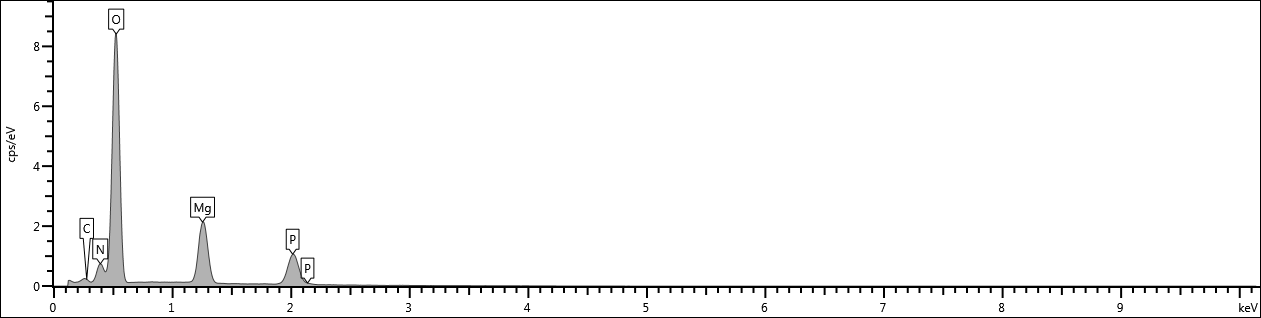

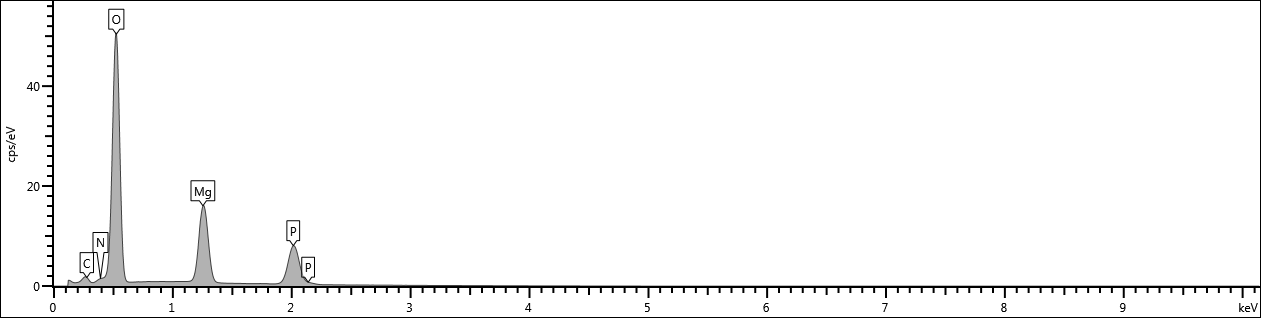
**

(B)

(A)

**Figure S2 (A) EDS analysis results for precipitants recovered from the MEC operation (Set C). (B) EDS analysis results for pure struvite (99.999% purity).**
