# Supplementary material for: Increasing phosphorus recovery from dewatering centrate in microbial electrolysis cells
Source: Biotechnol Biofuels. 2017 Mar 20;10:70. doi: 10.1186/s13068-017-0754-8 (PMC5359864; doi:10.1186/s13068-017-0754-8)
Supplement: Supplementary file 3 — Additional file 3: Figure S3 SEM images of struvite crystals on the SSM cathode after one fed-batch cycle (Set C). [file 13068_2017_754_MOESM3_ESM.docx]

 **

**

**Figure S3 SEM images of struvite crystals on the SSM cathode after one fed-batch cycle (Set C).**
